# Prognostic value of tricuspid regurgitation velocity and probability of pulmonary hypertension in patients undergoing transcatheter aortic valve implantation

**DOI:** 10.1007/s10554-017-1210-3

**Published:** 2017-07-01

**Authors:** Pawel Kleczynski, Artur Dziewierz, Agata Wiktorowicz, Maciej Bagienski, Lukasz Rzeszutko, Danuta Sorysz, Jaroslaw Trebacz, Robert Sobczynski, Marek Tomala, Dariusz Dudek

**Affiliations:** Institute of Cardiology, Jagiellonian University, Kopernika 17 Street, 31-501 Krakow, Poland

**Keywords:** Pulmonary hypertension, Aortic stenosis, Transcatheter aortic valve implantation, Mortality, Outcomes, Quality of life

## Abstract

Pulmonary hypertension (PH) is associated with adverse clinical outcomes after transcatheter aortic valve implantation (TAVI). We sought to investigate the effects of tricuspid regurgitant velocity (TRV) and echocardiographic probability of PH on clinical outcomes of patients undergoing TAVI. A total of 148 consecutive patients undergoing TAVI were included and stratified as having “low” (TRV ≤2.8 m/s), “intermediate” (TRV 2.9–3.4 m/s), and “high” (TRV >3.4 m/s) probability of PH. Only the patients from the “high” probability group were considered as patients with PH. All-cause mortality, complications rate and quality of life (QoL) were assessed according to VARC-2 recommendations. Of 148 patients, 65 (43.9%) were considered as patients with PH. These presented with higher NYHA class at baseline (p = 0.027) and had more frequently a history of previous stroke/transient ischemic attack (p = 0.019). A difference in all-cause mortality was noted at 12 months [PH (−) vs. PH (+): 9.6 vs. 21.5%; p = 0.043]; however, it was no longer significant after adjustment for age and gender (OR 2.39, 95% CI 0.91–6.24; p = 0.08). Unadjusted and adjusted rates of all-cause death at maximal follow-up of 13.3 (6.0–31.1) months were higher in patients with PH. However, the presence of PH was not identified as an independent predictor of all-cause mortality at follow-up. No difference in other complications rates and QoL were noted. The presence of TRV >3.4 m/s indicating “high” probability of PH may predict impaired clinical outcomes after TAVI. No impact of PH on QoL outcomes was confirmed.

## Introduction

Transcatheter aortic valve implantation (TAVI) is a less invasive than surgical aortic valve replacement (AVR) treatment modality for symptomatic severe aortic stenosis (AS). It is dedicated especially to elderly high-risk patients. Importantly, TAVI improves survival [1, 2] and quality of life (QoL) [3, 4] as compared to conservative treatment in inoperable patients. However, a successful TAVI procedure requires complex screening of patients, including detailed imaging as well as critical clinical assessment by an interdisciplinary Heart Team [5]. Longstanding AS results in pressure overload and subsequently concentric hypertrophy of the left ventricle with backward transmission of increased left ventricular and left atrial filling pressures. These as well as secondary pulmonary vasoconstriction and remodeling lead to secondary pulmonary hypertension (PH) and tricuspid regurgitation (TR). Importantly, PH has been shown to be associated with worse early and late outcomes after AVR [6, 7]. Thus PH is an important determinant of surgical risk and a significant component of the contemporary risk scores. However, the commonly used Society of Thoracic Surgeons score (STS) does not include PH as one of the risk factors during mortality assessment [8], whereas the EuroSCORE only considers the presence of severe PH with systolic pulmonary artery pressure (sPAP) >60 mm Hg. In addition, PH has been associated with short- and mid-term mortality after TAVI, but evidence is inconsistent [9, 10]. TAVI has been shown to decrease sPAP significantly [11, 12], a phenomenon associated with improved survival [13]. The recently updated European Society of Cardiology (ESC) and European Respiratory Society (ERS) guidelines on PH recommend using additional PH signs by assessing the ventricles, pulmonary artery, and inferior vena cava and right atrium in addition to the continuous wave Doppler measurement of the tricuspid regurgitant velocity (TRV) [14]. Thereby, PH probability is classified as “low”, “intermediate”, or “high”. We sought to investigate whether PH probability assessed by TRV only is a sufficient marker of clinical outcomes and QoL after TAVI.

## Materials and methods

A total of 148 consecutive patients who underwent TAVI at our center were included. All patients were diagnosed with symptomatic severe AS and had high surgical risk or contraindications for AVR. Patients were clinically evaluated to assess operative risk, comorbidities, frailty, and procedural feasibility. Baseline characteristics and procedural data were prospectively collected. Patient screening and selection were performed by a multidisciplinary Heart Team supported by clinical and imaging resources. As part of the pre-procedural work-up, all patients underwent a transthoracic echocardiogram before TAVI. According to the recently updated ESC/ERS guidelines for the diagnosis and management of PH [14], we assessed peak TRV for assigning the echocardiographic PH probability. Patients were stratified as having “low” (TRV ≤2.8 m/s), “intermediate” (TRV 2.9–3.4 m/s), and “high” (TRV >3.4 m/s) probability of PH. Only the patients from the “high” probability group were considered as patients with PH—the PH (+) group. Remaining patients with “low” or “intermediate” probability of PH were considered as patients without PH—the PH (−) group. TAVI procedures were done using Edwards Sapien, Edwards Sapien XT, Edwards Sapien 3 (Edwards Lifesciences), Medtronic CoreValve (Medtronic, Inc), JenaValve (JenaValve Technology), Lotus (Boston Scientific) and NVT (New Valve Technology). Access routes were transfemoral, transapical, subclavian, and direct aortic. Procedures were performed under general anesthesia or local anesthesia with sedation. All-cause mortality at 30 days, 12 months and maximal available follow-up was assessed. In addition, occurrence of other events and QoL was collected as recommended by the Valve Academic Research Consortium (VARC-2) [15]. QoL was assessed at baseline and at 12 months after TAVI with the validated Polish version of the EQ-5D-3L questionnaire including the visual analog scale (VAS) score. The study was approved by the institutional ethical board. Informed consent was obtained from all individual participants included in the study.

### Statistical analysis

Results are presented as number of patients (percentages) or median [interquartile range (IQR)] where applicable. Differences between groups were tested using Chi square test and the Fisher’s exact test for dichotomous variables and the Mann–Whitney *U* test for continuous variables. Changes in the proportions of patients who reported either “no problems” or “some problems”/“extreme problems” on the EQ-5D-3L between baseline and follow-up visits were analyzed using McNemar’s test. Differences in the VAS score between baseline and follow-up assessments were analyzed with a Wilcoxon signed-rank test. All paired comparisons between baseline and 12-month measurements were performed excluding unpaired results. The difference in mortality between groups during follow-up was assessed by the Kaplan–Meier method. In addition, differences in outcomes are presented as adjusted for age and gender odds ratios (OR) with 95% confidence intervals (CI). In addition, multivariable Cox regression analysis was performed to find significant predictors of all-cause mortality at maximal follow-up. All baseline characteristics and procedural data were tested. Forward selection with a probability value for covariates to enter the model was set at the 0.05 level. Results are presented as hazard ratios (HR) with 95% CI. Receiver-operating characteristic (ROC) curve analysis was used to assess the ability of TRV to predict death at 12 months and maximal available follow-up as well as the presence of reporting “some problems”/“extreme problems” on the EQ-5D-3L at baseline and 12 months. Results are presented as area under the ROC curve (AUC) with 95% CI. All tests were two-tailed, and a p value <0.05 was considered statistically significant. All statistical analysis was performed using SPSS 15.0 (SPSS, Inc., Chicago, IL, USA).

## Results

**Fig. 1:**
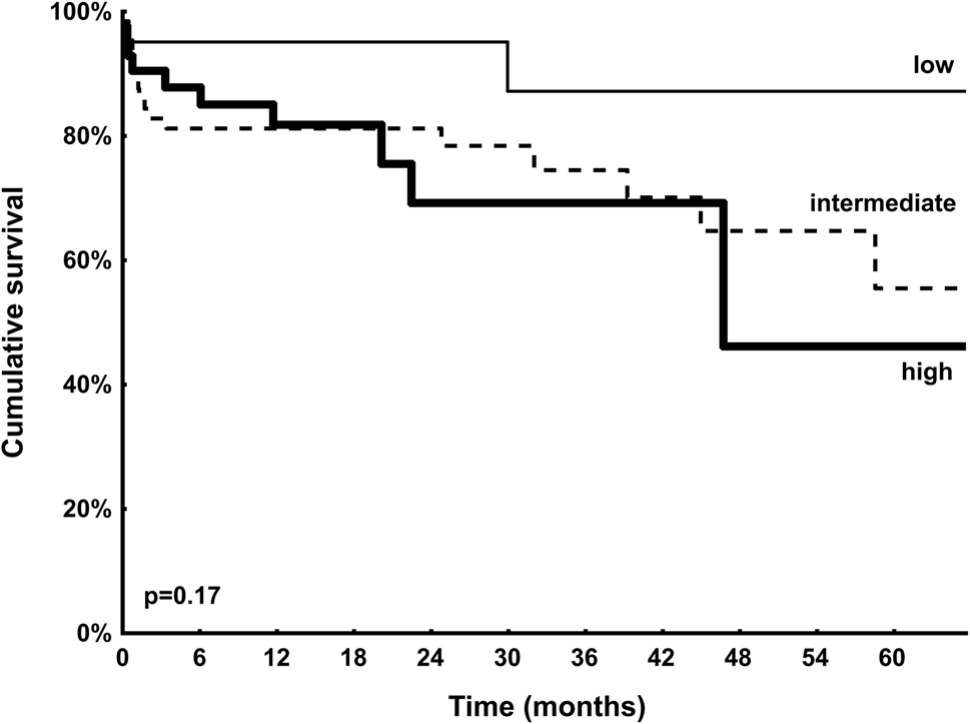
Kaplan–Meier curves for survival after transcatheter valve implantation stratified by echocardiographic pulmonary hypertension probability (low = *thin line*; intermediate = *dotted line*; high = *thick line*)

**Fig. 2:**
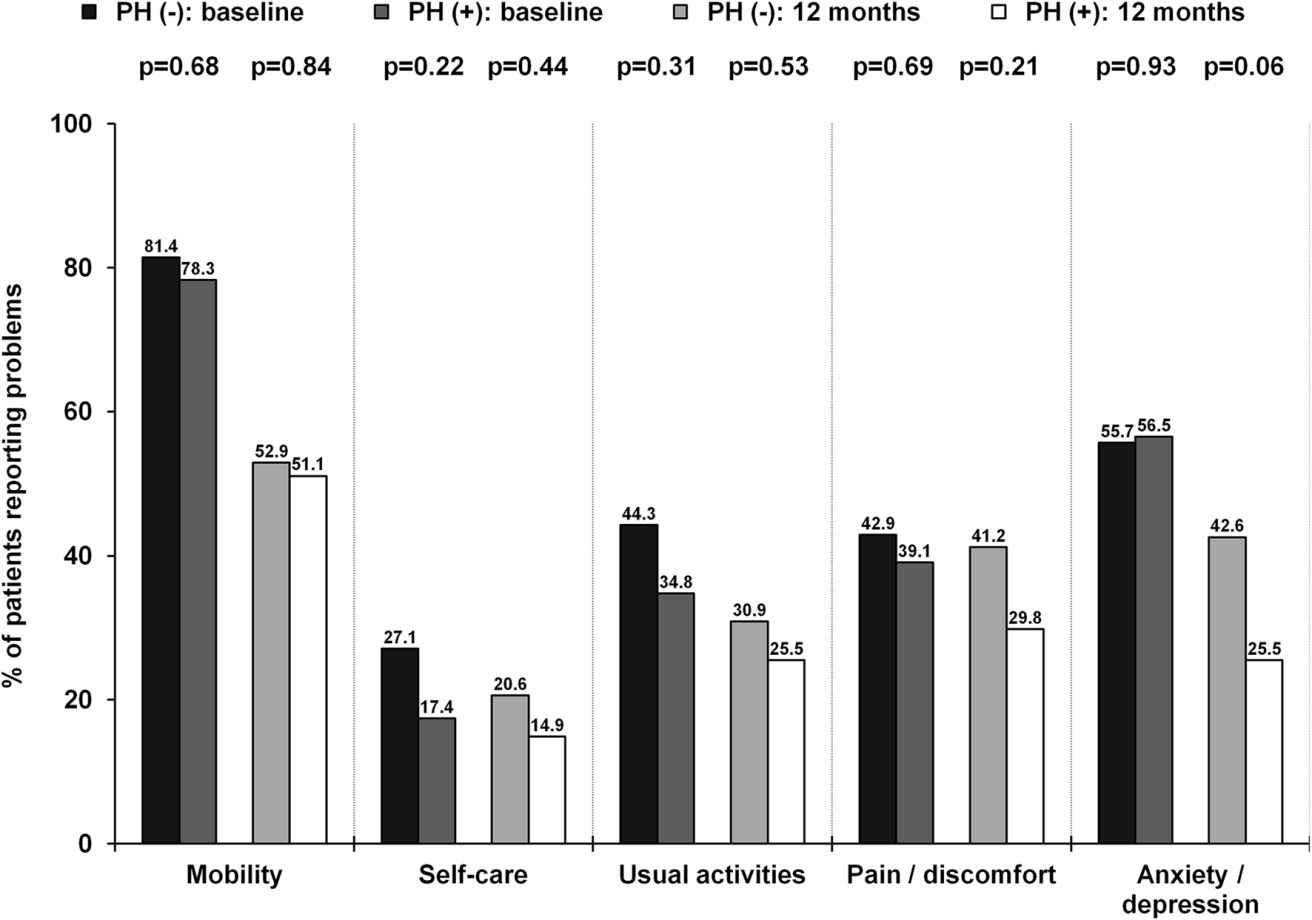
Proportions of patients that report either “some problems”/“extreme problems” for each category of the EQ-5D-3L at baseline and at 12 months

A total of 148 patients underwent elective TAVI. Median TRV was 3.3 (2.2–3.6) m/s. Of 148 patients, 65 (43.9%) patients had TRV >3.4 m/s and were considered as patients with PH. These patients presented with higher NYHA class at baseline (p = 0.027) and had more frequently a history of previous stroke/transient ischemic attack (p = 0.019). Remaining patients, 41 (27.7%) patients with TRV ≤2.8 m/s and 42 (28.4%) patients with TRV 2.9–3.4 m/s, were included in the PH (−) group. Detailed baseline clinical and procedural characteristics are shown in Tables 1 and 2. As shown in Table 3, in-hospital/30-day and 12-month complications rates were comparable between patients with and without PH. A difference in mortality was noted at 12 months [PH (−) vs. PH (+): 9.6 vs. 21.5%; p = 0.043]; however, it was no longer significant after adjustment for age and gender (OR 2.39, 95% CI 0.91–6.24; p = 0.08). On the contrary, unadjusted and adjusted rates of all-cause death at maximal follow-up of 13.3 (6.0–31.1) months were higher in patients with PH (Table 3). ROC curve analysis confirmed the association between TRV and all-cause mortality at 12 months (AUC 0.72, 95% CI 0.60–0.84; p = 0.001) and at maximal follow-up (AUC 0.71, 95% CI 0.61–0.80; p < 0.001). Presence of TRV >3.4 m/s was able to predict death at 12 months with 64% sensitivity and 62% specificity. The best performance was confirmed for cut-off value of 3.7 m/s which showed comparable sensitivity (60%) but much better specificity (82%). For maximal follow-up mortality, sensitivity was slightly lower with comparable results in terms of specificity (TRV >3.4 m/s: sensitivity 58%, specificity 63%; TRV >3.7 m/s: sensitivity 52%, specificity 84%). Importantly, PH was not identified as an independent predictor of mortality in multivariable Cox regression analysis. The only independent predictors were: incomplete coronary revascularization [HR (95% CI): 5.45 (2.38–12.52); p = 0.001], estimated glomerular filtration rate [HR (95% CI) per 1 ml/min/1.73 m^2^ increase: 0.96 (0.94–0.98); p = 0.001], and previous stroke/transient ischemic attack [HR (95% CI): 2.86 (1.17–7.00); p = 0.021]. Kaplan–Meier curves for survival after TAVI stratified by echocardiographic PH probability are shown in Fig. 1. Interestingly, we found that patients with “intermediate” probability of PH may have similar prognosis to those with “high” PH probability. All-cause 12-month mortality in “low”, “intermediate” and “high” PH probability groups was: 4.9 vs. 14.3 vs. 21.5%; p = 0.06, respectively. Similarly, all-cause mortality at maximum follow-up was: 7.3 vs. 23.8 vs. 30.8%; p = 0.018, respectively. No differences between groups in all components of EQ-5D-3L questionnaire were confirmed at 12 months (Fig. 2). The median VAS at baseline [PH (−) vs. PH (+): 40.0 (30.0–57.5) vs. 40.0 (35.0–50.0); p = 0.80] and 12 months after TAVI [70.0 (60.0–77.5) vs. 70.0 (62.5–80.0); p = 0.36] was comparable between groups. Similarly, no difference in VAS change during follow-up between both groups was reported [PH (−) vs. PH (+): 25.0 (10.0–37.5) vs. 25.0 (15.0–40.0); p = 0.47]. In ROC curve analysis we failed to confirm any association between TRV and the presence of reporting “some problems”/“extreme problems” on the EQ-5D-3L questionnaire at baseline and 12 months.

**Table 1:** Baseline characteristics and echocardiographic data

	All patients N = 148	PH (−) N = 83	PH (+) N = 65	P value
Age, median (IQR) (years)	82.0 (77.0–85.0)	82.0 (77.0–85.0)	82.0 (78.0–84.0)	0.94
Age ≥80 years, n (%)	92 (62.2)	49 (59.0)	43 (66.2)	0.38
Men, n (%)	56 (37.8)	28 (33.7)	28 (43.1)	0.25
Body mass index, median (IQR) (kg/m^2^)	27.2 (25.2–30.6)	27.2 (25.4–29.3)	27.6 (25.3–31.5)	0.42
eGFR, median (IQR) (ml/min/1.73 m^2^)	56.5 (40.0–72.0)	54.5 (42.0–72.0)	59.5 (37.0–72.0)	0.86
NYHA class, n (%)				0.027
I	0 (0.0)	0 (0.0)	0 (0.0)	
II	41 (27.7)	28 (33.7)	13 (20.0)	
III	97 (65.5)	47 (56.6)	50 (76.9)	
IV	10 (6.8)	8 (9.6)	2 (3.1)	
Arterial hypertension, n (%)	139 (93.9)	77 (92.8)	62 (95.4)	0.73
Diabetes mellitus, n (%)	48 (32.4)	23 (27.7)	25 (38.5)	0.17
Atrial fibrillation, n (%)	52 (35.1)	29 (34.9)	23 (35.4)	0.96
Previous MI, n (%)	48 (32.4)	28 (33.7)	20 (30.8)	0.70
Previous PCI, n (%)	43 (29.1)	21 (25.3)	22 (33.8)	0.26
Previous CABG, n (%)	28 (18.9)	19 (22.9)	9 (13.8)	0.16
CTO, n (%)	14 (9.5)	5 (6.0)	9 (13.8)	0.11
Incomplete revascularization, n (%)	22 (14.9)	9 (10.8)	13 (20.)	0.12
COPD, n (%)	19 (12.8)	8 (9.6)	11 (16.9)	0.19
Stroke/TIA, n (%)	17 (11.5)	5 (6.0)	12 (18.5)	0.019
Pacemaker, n (%)	17 (11.5)	8 (9.6)	9 (13.8)	0.43
Logistic euroscore I, median (IQR) (%)	14.5 (10.0–22.7)	14.5 (10.0–22.0)	14.5 (10.5–23.5)	0.45
STS, median (IQR) (%)	6.2 (4.0–17.3)	6.0 (4.0–14.8)	7.3 (4.8–21.0)	0.22
TG max, median (IQR) (mmHg)	86.0 (69.0–103.0)	85.0 (66.5–97.0)	88.0 (72.5–111.0)	0.15
TG mean, median (IQR) (mmHg)	50.0 (42.0–63.0)	49.0 (41.0–58.0)	50.0 (44.5–65.5)	0.14
AVA, median (IQR) [cm^2^]	0.7 (0.6–0.8)	0.7 (0.6–0.8)	0.6 (0.5–0.8)	0.06
LVEF, median (IQR) [%]	60.0 (50.0–65.0)	60.0 (48.0–65.0)	60.0 (50.0–65.0)	0.33
AR before, n (%)				0.70
0	48 (32.4)	30 (36.1)	18 (27.7)	
1	75 (50.7)	40 (48.2)	35 (53.8)	
2	20 (13.5)	10 (12.0)	10 (15.4)	
3	5 (3.4)	3 (3.6)	2 (3.1)	

AR = aortic regurgitation; AVA = aortic valve area; CABG = coronary artery bypass graft; COPD = chronic obstructive pulmonary disease; CTO = chronic total occlusion; DM = diabetes mellitus; eGFR = estimated glomerular filtration rate; IQR = interquartile range; LVEF = left ventricle ejection fraction; MI = myocardial infarction; NYHA = New York Heart Association; PCI = percutaneous coronary intervention; sPAP = systolic pulmonary artery pressure; STS = The Society of Thoracic Surgeons; TG = transaortic gradient; TIA = transient ischemic attack

**Table 2:** Procedural and echocardiographic data after the procedure

	All patients N = 148	PH (−) N = 83	PH (+) N = 65	P value
General anesthesia	98 (69.5)	53 (67.1)	45 (72.6)	0.48
Access type, n (%)				0.83
Transfemoral	117 (79.1)	66 (79.5)	51 (78.5)	
Transapical	28 (18.9)	16 (19.3)	12 (18.5)	
Transaortic	2 (1.4)	1 (1.2)	1 (1.5)	
Subclavian	1 (0.7)	0 (0.0)	1 (1.5)	
Device implanted, n (%)				0.41
Corevalve/Evolut R, n (%)	29 (19.6)	18 (21.7)	11 (16.9)	
Edwards Sapien	95 (64.2)	48 (57.8)	47 (72.3)	
Jena	10 (6.8)	7 (8.4)	3 (4.6)	
Lotus	9 (6.1)	7 (8.4)	2 (3.1)	
NVT	5 (3.4)	3 (3.6)	2 (3.1)	
Prosthesis size, n (%) (mm)				0.82
23	30 (20.3)	18 (21.7)	12 (18.5)	
25	8 (5.4)	5 (6.0)	3 (4.6)	
26	56 (37.8)	29 (34.9)	27 (41.5)	
27	8 (5.4)	5 (6.0)	3 (4.6)	
29	38 (25.7)	23 (27.7)	15 (23.1)	
31	8 (5.4)	3 (3.6)	5 (7.7)	
Prosthesis size, median (IQR) (mm)	26.0 (25.0–29.0)	26.0 (25.0–29.0)	26.0 (26.0–29.0)	0.74
TG max after TAVI, median (IQR) (mmHg)	13.0 (10.0–19.0)	12.8 (10.0–19.0)	15.0 (10.1–19.0)	0.21
TG mean after TAVI, median (IQR) (mmHg)	7.4 (5.1–10.0)	7.0 (5.0–10.5)	8.0 (6.0–10.0)	0.29
LVEF after, median (IQR) (%)	48.0 (41.0–55.0)	47.0 (40.0–50.0)	50.0 (45.0–60.0)	0.15
AR after, n (%)				0.041
0	84 (56.8)	45 (54.2)	39 (60.0)	
1	55 (37.2)	35 (42.2)	20 (30.8)	
2	7 (4.7)	1 (1.2)	6 (9.2)	
3	2 (1.4)	2 (2.4)	0 (0.0)	
Radiation dose, median (IQR) (mGy)	721.0 (632.5–827.5)	721.0 (634.0–826.0)	721.0 (631.0–823.0)	0.83
Contrast medium load, median (IQR) (ml)	75.0 (50.0–137.5)	75.0 (50.0–100.0)	75.0 (75.0–150.0)	0.07
Fluoroscopy time, median (IQR) (min)	13.0 (12.0–15.0)	13.0 (11.5–15.0)	13.0 (12.0–14.0)	0.62

*AR* aortic regurgitation, *LVEF* left ventricle ejection fraction, *TG* transaortic gradient

**Table 3:** Clinical outcomes

	PH (−) N = 83	PH (+) N = 65	P value	Adjusted OR (95% CI)*	Adjusted P value
In-hospital/30-day					
Bleeding	25 (30.1)	24 (36.9)	0.38	1.36 (0.680–2.72)	0.39
Blood transfusion	23 (27.7)	21 (32.3)	0.54	1.24 (0.61–2.54)	0.55
AKI grade 3	2 (2.4)	6 (9.2)	0.14	3.93 (0.76–20.47)	0.10
All-cause death	6 (7.2)	6 (9.2)	0.66	1.29 (0.39–4.22)	0.68
12-month					
Myocardial infarction	1 (1.2)	3 (4.6)	0.32	3.53 (0.35–35.92)	0.29
Stroke/TIA	3 (3.6)	7 (10.8)	0.11	2.93 (0.71–12.03)	0.14
New onset AF	3 (3.6)	7 (10.8)	0.11	2.77 (0.66–11.63)	0.16
New permanent pacemaker	15 (18.1)	9 (13.8)	0.49	0.74 (0.30–1.83)	0.52
All-cause death	8 (9.6)	14 (21.5)	0.043	2.39 (0.91–6.24)	0.08
Maximal follow-up					
All-cause death	13 (15.7)	20 (30.8)	0.028	2.26 (1.01–5.06)	0.047

*AF* atrial fibrillation, *AKI* acute kidney injury, *TIA* transient ischemic attack

^a^Derived from multivariable regression model—adjusted for age/gender

## Discussion

The major finding of our study is that patients with severe AS and PH who undergone TAVI demonstrated higher long-term mortality than patients without PH but with comparable complications rate and QoL outcomes. Our findings are in line with results of the previous studies showing that PH is associated with an increased mortality after TAVI [6, 9–13, 16–18]. However, our study indicate that the outcomes of patients with “intermediate” probability of PH might not be as good as expected and even similar to patients with “high” PH probability.

PH is observed quite frequent in patients with AS, as exercise induced PH is present in over half of the patients with severe asymptomatic AS [19]. Among patients undergoing TAVI, concomitant PH on echocardiography is found in 20–75% [6, 9–13, 16–18, 20]. However, different PH definitions and cut-off values may cause some inconsistence in assessing PH frequency. Importantly, exercise induced PH increased cardiac events in patients with severe asymptomatic AS [19]. Also, in patients undergoing surgical AVR for AS, baseline PH and its severity are associated with mortality, serious complications, and worse late survival [21–24]. Thus, patients with very elevated sPAP are often disqualified from surgical valve replacement due to concerns about high peri-operative morbidity and mortality or doubts about whether or not valve replacement will provide any clinical benefit. Data on the impact of PH on outcomes after TAVI are less consistent. In the study by Lindman et al. increased sPAP were associated with increased mortality, repeat hospitalizations, and strokes during the first year after TAVI [25]. Barbash et al. [9] showed that the presence of sPAP >50 mmHg on echocardiography increased the mortality rate immediately after TAVI. In addition, patients with sPAP >50 mmHg had a prolonged hospitalization at the intensive care unit. Also, another studies have concluded a higher mortality rate at 12 months among patients with PH [16–18]. On the contrary, in the FRANCE-2 registry [18], the 30-day outcome did not differ among 2435 TAVI patients with sPAP <40, 40–60, and ≥60 mmHg as assessed by echocardiography. Similarly, Tamburino et al. [26] did not identify sPAP >60 mmHg on echocardiography in TAVI patients as an independent predictor of 30-day mortality. However, in the recent meta-analysis by Tang et al. on 9204 patients with severe AS undergoing TAVI, baseline PH was associated with increased 30-day and 1-year cardiovascular mortality as well as 1- and 2-year all-cause mortality [27].

Pulmonary artery pressures tend to decrease after AVR and after TAVI. However, some patients with severe pre-operative PH have a persistent severe postprocedural elevation in sPAP, which is associated with a higher mortality than in patients with the decrease in sPAP [13]. TAVI was found to be beneficial in decreasing sPAP [10, 16, 17]. Importantly, Sinning et al. found that in 16 patients with remaining high sPAP (>60 mmHg) at 3 months after TAVI, 2-year mortality was higher as compared to 226 patients with sPAP reduced ≤60 mmHg [13]. It might suggest that both decrease in sPAP and PH severity during follow-up might impact the prognosis of patients after TAVI. Also, surgical data have demonstrated that the reoccurrence of preoperative PH is usually detected during longer follow-up of 3–4 years [28]. These may emphasize the value of longer follow-up in patients with baseline PH undergoing TAVI.

As mentioned, the frequency and assessed severity of PH may strongly depend on the definition used. Nijenhuis et al. found that [10] the new echocardiographic PH probability model incorporating additional PH signs performs well as a discriminator for prognosis after TAVI, irrespective of other patient and procedural characteristics. Patients with a “high” PH probability are at increased risk of early and late mortality compared to “low” and “intermediate” PH probability groups [10]. In our study, the probability of PH was estimated based on TRV only. However, it should be stressed that even this simplified approach of the assessment of PH probability was sufficient to identify patients with elevated risk of death at follow-up. Also, TRV was able to discriminate patients with “intermediate” probability of PH whose may have similar prognosis to those with “high” PH probability. Probably, the assessment of additional PH signs might be particularly important for further evaluation and prediction of outcomes. In the study by Nijenhuis there were no differences in the occurrence or severity of a paravalvular leak, conduction disturbances, and the rate of thrombo-embolic or bleeding complications [10]. Compared to the “low” and “intermediate” PH probability, a “high” PH probability was more often associated with acute kidney injury, delirium, and prolonged hospitalization [10]. On the contrary, no difference.

Evaluation of QoL seems to be an important index as frequently not a reduction in mortality but improvement in daily life comfort is most desirable by patients themselves [3, 4]. Importantly, patients with PH had a reduced QoL as compared to the general population [28]. Also, a decrease in QoL was shown to be a predictor of worse outcomes in adult patients with PH due to congenital heart disease [25911012]. On the contrary, the impact of PH on QoL in patients undergoing TAVI has not been tested so far. Amelioration of QoL after TAVI was presented in recently published studies [3, 4]. The improvement in QoL after TAVI may be higher than observed after SAVR, even with the use of minimally-invasive surgical techniques (mini-thoracotomy, mini-sternotomy) [29]. In our study no differences in QoL were observed between groups. This might suggest equal response to TAVI in terms of QoL regardless of PH presence.

### Study limitations

The presented study has several limitations. The most important is a single-center, prospective non-randomized observational design of the study. A relatively small cohort of included patients and the size of the two main groups have not allowed us for definitive confirmation/exclusion of the relationship between PH status and clinical outcomes of patients after TAVI. Right heart catheterization was not performed, limiting control for invasive measurements. Unfortunately, data on the PH assessment during follow-up were not available in all patients, thus limiting the possibility of correction for PH improvement. On the other hand, this study represents a comprehensive analysis of consecutive patients without any exclusion criteria and complete assessment of QoL.

## Conclusions

The presence of TRV >3.4 m/s indicating “high” probability of PH may predict impaired clinical outcomes after TAVI. No impact of PH on QoL outcomes was confirmed.

## References

[1] Leon MB, Smith CR, Mack M, Miller DC, Moses JW, Svensson LG, Tuzcu EM, Webb JG, Fontana GP, Makkar RR, Brown DL, Block PC, Guyton RA, Pichard AD, Bavaria JE, Herrmann HC, Douglas PS, Petersen JL, Akin JJ, Anderson WN, Wang D, Pocock S, PARTNER Trial Investigators (2010). Transcatheter aortic-valve implantation for aortic stenosis in patients who cannot undergo surgery. N Engl J Med.

[2] Bagienski M, Kleczynski P, Dziewierz A, Rzeszutko L, Sorysz D, Trebacz J, Sobczynski R, Tomala M, Stapor M, Gackowski A, Dudek D (2016). Early and mid-term outcomes after transcatheter aortic valve implantation. Data from a single center registry. Adv Interv Cardiol.

[3] Kleczyński P, Bagieński M, Sorysz D, Rzeszutko L, Trębacz J, Tomala M, Sobczyński R, Dziewierz A, Surdacki A, Dudek D (2014). Short- and intermediate-term improvement of patient quality of life after transcatheter aortic valve implantation: a single-center study. Kardiol Pol.

[4] Kleczyński P, Bagieński M, Dziewierz A, Rzeszutko Ł, Sorysz D, Trębacz J, Sobczyński R, Tomala M, Stąpór M, Dudek D (2016). Twelve-month quality of life improvement and all-cause mortality in elderly patients undergoing transcatheter aortic valve replacement. Int J Artif Organs.

[5] Vahanian A, Alfieri O, Andreotti F, Antunes MJ, Barón-Esquivias G, Baumgartner H, Borger MA, Carrel TP, De Bonis M, Evangelista A, Falk V, Iung B, Lancellotti P, Pierard L, Price S, Schäfers HJ, Schuler G, Stepinska J, Swedberg K, Takkenberg J, Von Oppell UO, Windecker S, Zamorano JL, Zembala M, Joint Task Force on the Management of Valvular Heart Disease of the European Society of Cardiology (ESC), European Association for Cardio-Thoracic Surgery (EACTS) (2012). Guidelines on the management of valvular heart disease (version 2012). Eur Heart J.

[6] Melby SJ, Moon MR, Lindman BR, Bailey MS, Hill LL, Damiano RJ (2011). Impact of pulmonary hypertension on outcomes after aortic valve replacement for aortic valve stenosis. J Thorac Cardiovasc Surg.

[7] McLaughlin VV, Archer SL, Badesch DB, Barst RJ, Farber HW, Lindner JR, Mathier MA, McGoon MD, Park MH, Rosenson RS, Rubin LJ, Tapson VF, Varga J, American College of Cardiology Foundation Task Force on Expert Consensus Documents, American Heart Association (2009). ACCF/AHA 2009 expert consensus document on pulmonary hypertension a report of the American College of Cardiology Foundation Task Force on expert consensus documents and the American Heart Association developed in collaboration with the American College of Chest Physicians; American Thoracic Society, Inc.; and the Pulmonary Hypertension Association. J Am Coll Cardiol.

[8] Brown JM, O’Brien SM, Wu C, Sikora JA, Griffith BP, Gammie JS (2009). Isolated aortic valve replacement in North America comprising 108, 687 patients in 10 years: changes in risks, valve types, and outcomes in the Society of Thoracic Surgeons National Database. J Thorac Cardiovasc Surg.

[9] Barbash IM, Escarcega RO, Minha S, Ben-Dor I, Torguson R, Goldstein SA, Wang Z, Okubagzi P, Satler LF, Pichard AD, Waksman R (2015). Prevalence and impact of pulmonary hypertension on patients with aortic stenosis who underwent transcatheter aortic valve replacement. Am J Cardiol.

[10] Nijenhuis VJ, Huitema MP, Vorselaars VM, Swaans MJ, de Kroon T, van der Heyden JA, Rensing BJ, Heijmen R, Ten Berg JM, Post MC (2016). Echocardiographic pulmonary hypertension probability is associated with clinical outcomes after transcatheter aortic valve implantation. Int J Cardiol.

[11] Ben-Dor I, Goldstein SA, Pichard AD, Satler LF, Maluenda G, Li Y, Syed AI, Gonzalez MA, Gaglia MA, Wakabayashi K, Delhaye C, Belle L, Wang Z, Collins SD, Torguson R, Okubagzi P, Aderotoye A, Xue Z, Suddath WO, Kent KM, Epstein SE, Lindsay J, Waksman R (2011). Clinical profile, prognostic implication, and response to treatment of pulmonary hypertension in patients with severe aortic stenosis. Am J Cardiol.

[12] Müller S, Velik-Salchner C, Edlinger M, Bonaros N, Heinz A, Feuchtner G, Bartel T (2016). Intracardiac Doppler echocardiography for monitoring of pulmonary artery pressures in high-risk patients undergoing transcatheter aortic valve replacement. J Am Soc Echocardiogr.

[13] Sinning JM, Hammerstingl C, Chin D, Ghanem A, Schueler R, Sedaghat A, Bence J, Spyt T, Werner N, Kovac J, Grube E, Nickenig G, Vasa-Nicotera M (2014). Decrease of pulmonary hypertension impacts on prognosis after transcatheter aortic valve replacement. EuroIntervention.

[14] Galiè N, Humbert M, Vachiery JL, Gibbs S, Lang I, Torbicki A, Simonneau G, Peacock A, Vonk Noordegraaf A, Beghetti M, Ghofrani A, Gomez Sanchez MA, Hansmann G, Klepetko W, Lancellotti P, Matucci M, McDonagh T, Pierard LA, Trindade PT, Zompatori M, Hoeper M, Aboyans V, Vaz Carneiro A, Achenbach S, Agewall S, Allanore Y, Asteggiano R, Paolo Badano L, Albert Barberà J, Bouvaist H, Bueno H, Byrne RA, Carerj S, Castro G, Erol Ç, Falk V, Funck-Brentano C, Gorenflo M, Granton J, Iung B, Kiely DG, Kirchhof P, Kjellstrom B, Landmesser U, Lekakis J, Lionis C, Lip GY, Orfanos SE, Park MH, Piepoli MF, Ponikowski P, Revel MP, Rigau D, Rosenkranz S, Völler H, Luis Zamorano J (2016). 2015 ESC/ERS Guidelines for the diagnosis and treatment of pulmonary hypertension: the Joint Task Force for the Diagnosis and Treatment of Pulmonary Hypertension of the European Society of Cardiology (ESC) and the European Respiratory Society (ERS): endorsed by: Association for European Paediatric and Congenital Cardiology (AEPC), International Society for Heart and Lung Transplantation (ISHLT). Eur Heart J.

[15] Kappetein AP, Head SJ, Généreux P, Piazza N, van Mieghem NM, Blackstone EH, Brott TG, Cohen DJ, Cutlip DE, van Es GA, Hahn RT, Kirtane AJ, Krucoff MW, Kodali S, Mack MJ, Mehran R, Rodés-Cabau J, Vranckx P, Webb JG, Windecker S, Serruys PW, Leon MB, Valve Academic Research Consortium-2 (2012). Updated standardized endpoint definitions for transcatheter aortic valve implantation: the Valve Academic Research Consortium-2 consensus document. EuroIntervention.

[16] O’Sullivan CJ, Wenaweser P, Ceylan O, Rat-Wirtzler J, Stortecky S, Heg D, Spitzer E, Zanchin T, Praz F, Tüller D, Huber C, Pilgrim T, Nietlispach F, Khattab AA, Carrel T, Meier B, Windecker S, Buellesfeld L (2015). Effect of pulmonary hypertension hemodynamic presentation on clinical outcomes in patients with severe symptomatic aortic valve stenosis undergoing transcatheter aortic valve implantation: insights from the new proposed pulmonary hypertension classification. Circ Cardiovasc Interv.

[17] Schewel D, Schewel J, Martin J, Voigtländer L, Frerker C, Wohlmuth P, Thielsen T, Kuck KH, Schäfer U (2015). Impact of transcatheter aortic valve implantation (TAVI) on pulmonary hyper-tension and clinical outcome in patients with severe aortic valvular stenosis. Clin Res Cardiol.

[18] Luçon A, Oger E, Bedossa M, Boulmier D, Verhoye JP, Eltchaninoff H, Iung B, Leguerrier A, Laskar M, Leprince P, Gilard M, Le Breton H (2014). Prognostic implications of pulmonary hypertension in patients with severe aortic stenosis undergoing transcatheter aortic valve implantation: study from the FRANCE 2 Registry. Circ Cardiovasc Interv.

[19] Lancellotti P, Magne J, Donal E, O’Connor K, Dulgheru R, Rosca M, Pierard LA (2012). Determinants and prognostic significance of exercise pulmonary hypertension in asymptomatic severe aortic stenosis. Circulation.

[20] Stokłosa P, Szymański P, Dąbrowski M, Zakrzewski D, Michałek P, Orłowska-Baranowska E, El-Hassan K, Chmielak Z, Witkowski A, Hryniewiecki T (2015). The impact of transcatheter aortic valve implantation on left ventricular performance and wall thickness - single-centre experience. Postepy Kardiol Interwencyjnej.

[21] Roselli EE, Abdel Azim A, Houghtaling PL, Jaber WA, Blackstone EH (2012). Pulmonary hypertension is associated with worse early and late outcomes after aortic valve replacement: implications for transcatheter aortic valve replacement. J Thorac Cardiovasc Surg.

[22] Zuern CS, Eick C, Rizas K, Stoleriu C, Woernle B, Wildhirt S, Herdeg C, Stock U, Gawaz M, Bauer A (2012). Prognostic value of mild-to-moderate pulmonary hyper-tension in patients with severe aortic valve stenosis undergoing aortic valve replacement. Clin Res Cardiol.

[23] Oliveira SM, Correia AS, Paiva M, Gonc¸alves A, Pereira M, Alves E, Dias P, Almeida R, Abreu A, Pinho P (2012). Long-term survival, autonomy, and quality of life of elderly patients undergoing aortic valve replacement. J Card Surg.

[24] Sundt TM, Bailey MS, Moon MR, Mendeloff EN, Huddleston CB, Pasque MK, Barner HB, Gay WA (2000). Quality of life after aortic valve replacement at the age of >80 years. Circulation.

[25] Lindman BR, Zajarias A, Maniar HS, Miller DC, Suri RM, Arnold SV, Webb J, Svensson LG, Kodali S, Xu K, Ayele GM, Lin F, Wong SC, Babaliaros V, Thourani VH, Douglas PS, Lim S, Leon MB, Mack MJ (2015). Risk stratification in patients with pulmonary hypertension undergoing transcatheter aortic valve replacement. Heart.

[26] Tamburino C, Capodanno D, Ramondo A, Petronio AS, Ettori F, Santoro G, Klugmann S, Bedogni F, Maisano F, Marzocchi A, Poli A, Antoniucci D, Napodano M, De Carlo M, Fiorina C, Ussia GP (2011). Incidence and predictors of early and late mortality after transcatheter aortic valve implantation in 663 patients with severe aortic stenosis. Circulation.

[27] Tang M, Liu X, Lin C, He Y, Cai X, Xu Q, Hu P, Gao F, Jiang J, Lin X, Zhu Q, Wang L, Kong H, Yu Y, Wang J (2017). Meta-analysis of outcomes and evolution of pulmonary hypertension before and after transcatheter aortic valve implantation. Am J Cardiol.

[28] Guillevin L, Armstrong I, Aldrighetti R, Howard LS, Ryftenius H, Fischer A, Lombardi S, Studer S, Ferrari P (2013). Understanding the impact of pulmonary arterial hypertension on patients’ and carers’ lives. Eur Respir Rev.

[29] Tokarek T, Siudak Z, Dziewierz A, Sobczyński R, Zasada W, Sorysz D, Olszewska-Wityńska K, Bryniarski K, Krawczyk-Ożóg A, Żabówka A, Sadowski J, Dudek D (2016). Assessment of quality of life in patients after surgical and transcatheter aortic valve replacement. Catheter Cardiovasc Interv.

